# Editorial: Unveiling the structure and function of brain microcircuits: Experiments, algorithms and simulations

**DOI:** 10.3389/fncir.2022.991137

**Published:** 2022-08-12

**Authors:** Guanxiao Qi, Junsong Zhang, Alexander D. Bird

**Affiliations:** ^1^Institute of Neuroscience and Medicine 10 (INM-10), Research Centre Jülich, Jülich, Germany; ^2^Department of Artificial Intelligence, School of Informatics, Xiamen University, Xiamen, China; ^3^Ernst Strüngmann Institute for Neuroscience, Frankfurt am Main, Germany; ^4^Interdisciplinary Centre for 3Rs in Animal Research (ICAR3R), Faculty of Medicine, Justus-Liebig-University, Giessen, Germany; ^5^Frankfurt Institute for Advanced Studies, Frankfurt am Main, Germany

**Keywords:** neuron, synapse, electrophysiology, morphology, simulation

## Introduction

The brain is one of the most complex systems known to man and its components span several orders of magnitude ranging from the nano- to centimeter level (Lichtman and Denk, 2011). To understand the working principles of the different components of the brain, it is necessary to unveil the structure, function and interactions of its building blocks - neuronal microcircuits (Luo, 2021). These are groups of synaptically connected cells that are responsible for neuronal signal processing, integration, and coordination. In the last decades, experimental studies of neuronal microcircuits using many different techniques, such as paired (multiple) intracellular recordings, *in vivo* and *in vitro* calcium imaging, optogenetics, super-resolution light microscopy, tissue clearing, viral tracing, and electron microscopy, have greatly expanded our knowledge of the connectivity rules and dynamical properties in different neural systems (Alivisatos et al., 2013; Lerner et al., 2016; Feldmeyer et al., 2020). In parallel, computer simulations of biophysically realistic neuronal network models such as the Blue Brain Project (Markram et al., 2015) and the Allen Brain Modeling ToolKit (Dai et al., 2020) have deepened our understanding of the brain and provided a testbed for new theories and hypotheses. However, a full understanding of synaptic signal processing and neuronal computation at the level of neuronal microcircuits remains an open and important question. In addition to experimental approaches, new challenges and opportunities arise in the development of sophisticated analysis algorithms and large-scale neuronal simulations which are indispensable to uncover the mysteries of the brain microcircuits.

## Papers in this collection

In this Frontiers Research Topic, we have put together two review articles and two method articles (see below).

Thomson provides a historical perspective of more than a century of dedicated research on synaptic transmission and neuronal circuits. She gives us a deep insight into the hypotheses and controversies in the study of circuits and synapses which include the “neuron doctrine” vs. the “reticular concept,” the chemical or electrical synaptic transmission and the search for neurotransmitter types among other topics. She also discusses in which way scientific results could be obtained, how to formulate hypotheses and how to avoid turning popular theories into dogma that may stifle future research.

Xu et al. review the neural circuits for social interactions, such as social exploration, social hierarchy, social memory, and social preference, at the molecular, cellular, and network levels. They provide a broad view of how multiple microcircuits and input-output circuits converge on the medial prefrontal cortex, hippocampus, and amygdala to regulate complex social behaviors, as well as a potential novel view for better control over pathological development.

Li et al. designed Bitbow, a digital format of Brainbow which exponentially expands the color palette to provide tens of thousands of spectrally resolved unique labels. As a proof of principle, they generated transgenic Bitbow *Drosophila* lines, established statistical tools, and streamlined sample preparation, image processing, and data analysis pipelines to conveniently map neural lineages, study neuronal morphology and reveal neural network patterns.

Wilson et al. present a straightforward and very useful platform by which patterns of electricity can be arbitrarily defined and distributed across a brain circuit, either simultaneously, asynchronously, or in complex patterns that can be easily designed and orchestrated with precise timing. Interfacing with acute slices of mouse cortex, they show that the system can be used to activate neurons at many locations and drive synaptic transmission in distributed patterns, and that this elicits new forms of plasticity at the circuit level.

We hope that this collection of papers will stimulate more studies in future on experiments, algorithms and simulations for exploring microcircuit structure and function, which is an important step toward decoding the healthy and diseased brain.

## Author contributions

GQ, JZ, and AB wrote the manuscript. All authors contributed to the article and approved the submitted version.

## Funding

GQ was supported by the Helmholtz Society. JZ was supported by the National Nature Science Foundation of China (No. 61772440) and the Project of Industry-University-Research Collaborative Innovation in Fujian Province Universities (No. 2022Y4004). AB was supported by BMBF (No. 031L0229) and DFG (JE 528/10-1).

## Conflict of interest

The authors declare that the research was conducted in the absence of any commercial or financial relationships that could be construed as a potential conflict of interest.

## Publisher's note

All claims expressed in this article are solely those of the authors and do not necessarily represent those of their affiliated organizations, or those of the publisher, the editors and the reviewers. Any product that may be evaluated in this article, or claim that may be made by its manufacturer, is not guaranteed or endorsed by the publisher.
